# Chemoprophylaxis trial designs in epidemics: insights from a systematic review of COVID-19 study registrations

**DOI:** 10.1186/s13063-021-05323-4

**Published:** 2021-05-29

**Authors:** Lakshmi Manoharan, Piero Olliaro, Peter W. Horby, Conall H. Watson

**Affiliations:** 1 Centre for Tropical Medicine and Global Health, Nuffield Department of Medicine University of Oxford, Old Road Campus, Roosevelt Dr, Oxford, OX3 7LG UK; 2 International Severe Acute Respiratory and Emerging Infection Consortium (ISARIC) Global Support Centre, Centre for Tropical Medicine and Global Health, Nuffield Department of Medicine University of Oxford, Old Road Campus, Roosevelt Dr, Oxford, OX3 7LG UK

**Keywords:** Chemoprophylaxis, Coronavirus, SARS-CoV-2, COVID-19, Systematic review, Adaptive design, Hydroxychloroquine, Chloroquine

## Abstract

**Background:**

Chemoprophylactics against emerging epidemic and pandemic infectious diseases offer potential for prevention but require efficacy and safety analysis before widespread use can be recommended. Chemoprophylaxis with repurposed drugs enables deployment ahead of development of novel vaccines. It may have particular utility as a stopgap ahead of vaccine deployment or when vaccines become less effective on virus variants, in countries where there may be structural inaccessibility to vaccines or in specific risk-groups.

Rapid implementation of robust trial designs is a persistent challenge in epidemics. We systematically reviewed SARS-CoV-2 and COVID-19 chemoprophylaxis trial registrations from the first 21 weeks of the pandemic to critically appraise significant design features and alignment of study populations to clinical and public health uses, and describe candidate chemoprophylactic agents.

**Methods:**

We searched online international trial databases from 31 Dec 2019 to 26 May 2020 using keywords “proph*” or “prevention”. Trial protocols assessing efficacy of chemoprophylactic agents for COVID-19 were included. Trial components were screened for eligibility and relevant studies extracted. Key trial design features were assessed.

**Results:**

We found 76 chemoprophylaxis study registrations, proposing enrolment of 208,367 people with median size of 490 (IQR 262–1710). A randomised design was specified for 63 trials, 61 included a control group and total proposed enrolment size was 197,010, median 600 (IQR 236–1834).

Four protocols provided information on effect size sought. We estimate that for a control group attack rate of 10%, 66% of trials would be underpowered to detect a 50% effect size, and 97% of trials would be underpowered to detect a 30% effect size (at the 80% level). We found evidence of adaptive design in one trial registration only. Laboratory-confirmed infection with or without symptoms was the most common primary outcome. Polymerase chain reaction testing alone was used in 46% of trials, serological testing in 6.6% and 14.5% used both testing methods.

Healthcare workers were the target population in 52/79 (65.8%) trials: 49 pre-exposure prophylaxis (PrEP) and 3 post-exposure prophylaxis (PEP). Sixteen trials (20.3%) planned PEP in close contacts. Five studies (6.3%) considered chemoprophylaxis in clinical-risk patients. Older adults were the focus of recruitment in only 3 (3.8%) studies (all long-term care facilities). Two (2.5%) studies of PrEP in the general population included older adults. Hydroxychloroquine was the most common candidate agent in 55/79 trials (69.6%), followed by chloroquine (4/79, 5.0%) and lopinavir/ritonavir (3/79, 3.8%).

**Conclusion:**

Many registered COVID-19 chemoprophylaxis efficacy trials were underpowered to detect clinically meaningful protection at epidemiologically informed attack rates. This, compounded with the time that has taken to organise these trials as compared to the rapid development of COVID-19 vaccines, has rendered these trials of marginal importance. International coordination mechanisms and collaboration is required. Supporting the design of feasible chemoprophylaxis trials, large enough to generate strong evidence, early on in an epidemic using adaptive platform trial designs will allow structured entry and exit of candidate agents and rapid stand-up of trial infrastructure.

**Review protocol registration:**

Our protocol is registered at https://www.osf.io/vp56f on May 20, 2020.

**Supplementary Information:**

The online version contains supplementary material available at 10.1186/s13063-021-05323-4.

## Background

Chemoprophylaxis can be defined as the administration of non-vaccine pharmaceuticals to a person not known to be infected to prevent infection and/or the health and disease consequences of infection. Deployed within broader public health prevention strategies, effective agents could lower the burden of emerging, epidemic and pandemic diseases such as COVID-19 (caused by severe acute respiratory syndrome coronavirus 2, SARS-CoV-2) [1, 2]. Efficacy and safety needs to be tested through trials aligned to the potential uses and epidemiological needs for chemoprophylaxis.

While epidemic control relies heavily on vaccine availability, it generally takes time to develop vaccines against a new virus. Chemoprophylaxis may have particular utility as a stopgap ahead of vaccine deployment or should vaccines become less effective on virus variants. They may also be of use in countries with structural inaccessibility leading to delays in vaccine deployment or in specific risk-groups.

In clinical practice, anti-infective chemoprophylaxis is typically classified into pre-exposure prophylaxis (PrEP) and post-exposure prophylaxis (PEP). PrEP has disadvantages with respect to vaccination, as it provides time-limited protection and requires administration of the agent throughout the perceived period of risk of infection — e.g. reflecting sessional clinical exposure, epidemic transmission or endemic seasonal patterns. PEP of contacts may follow identification of a case in a household, nursing home or following a close-contact encounter without effective personal protective equipment (PPE) in community or clinical settings.

Where vaccines are not available, due to scientific development timelines or constrained accessibility of approved products, effective chemoprophylactics could address the preventative-pharmaceutical gap. In a pandemic, this might be through repurposing of licenced pharmaceuticals shown efficacious in rapid trials ahead of novel vaccines being evaluated, taking advantage of established safety records to bypass phase I and IIa studies. In low- and middle- income countries, financial and structural barriers to novel vaccines are a particular concern, alongside the potentially greater propensity for disease emergence [3–5]. Effective chemoprophylaxis against coronaviruses at the zoonotic interface could be a further use, similar to neuraminidase inhibitors in humans potentially exposed to avian influenza. Influenza antivirals also show the role of chemoprophylaxis where there is imperfect vaccine efficacy and viral genetic changes.

Candidate chemoprophylactics require evaluation of potential harms, particularly for mass drug administration and for potential interactions with concomitant medication. For example, amongst the early COVID-19 chemoprophylaxis candidates, Torsades de Pointes arrhythmias associated with hydroxychloroquine (HCQ) [6–8] and cytochrome P450 interactions by lopinavir-ritonavir deserve specific consideration. There is greater potential for drug interactions and adverse events in the old, frail and multi-morbid [9]. These potential harms warrant careful evaluation through well-designed studies which are large enough not only to assess efficacy but also to capture uncommon adverse events if established safety profiles do not exist in relevant populations. As evidence about candidate interventions accrues within or outside a study, adaptive or platform trials may offer utility by readily and rapidly enabling adjustment of trial arms.

The generation of robust and reliable data that can be translated into clinical use requires that chemoprophylaxis trials in epidemics be adequately powered, and that choices in trial design, study population and drug candidate reflects clinical and public health need while accounting for emerging epidemiology.

Trials started early in epidemics should have a better chance of reaching conclusions than those started after cases peak [10]. Accordingly, we undertook a systematic review of published trial protocols from the first 21 weeks of the pandemic to assess these characteristics and evaluate the COVID-19 chemoprophylactic trial landscape.

## Methods

We searched the World Health Organisation (WHO) International Clinical Trial Registry Protocol (ICTRP) database of COVID-19 trials from 31 Dec 2019 to 26 May 2020 using the keywords “proph*” or “prevention”. This search was augmented by screening additional international trial registries. ClinicalTrials.gov, the EU Clinical Trials Register and the Chinese Clinical Trial Registry was searched using (proph* or prevention) and (“COVID-19” or “coronavirus”). We searched National Institute of Public Health of Japan (NIPH) and the Pan African Clinical Trial Registry (PACTR) using “COVID-19” or “coronavirus” to maximise search results as key words could not be combined. Trial protocols in any population which assessed chemoprophylactic agents for COVID-19 were included, and protocols were machine translated into English when necessary. Protocols for phase I and early phase II trials and observational trials were excluded to ensure only trials assessing agent efficacy were included. Titles and trial components were screened for eligibility by a single reviewer (LM) and relevant studies extracted. Key trial design features were assessed, based on the information available from published protocols. Key features extracted were characteristics of recruited target populations and estimated sample sizes, candidate chemoprophylactic agents and dose regimens, duration of treatment and follow-up, primary and key secondary outcomes and measurement methods, effect sizes sought and use of a platform or adaptive design. We defined a chemoprophylaxis megatrial as one containing at least 10,000 participants [11]. We estimated the sample sizes required to detect a range of effect sizes at different attack rates and compared these to registered trial sizes. Data extraction and synthesis was performed using Microsoft Excel. Analyses including power calculations were performed in R (3.6.3), using estimated sample size from protocols, power and effect sizes when provided and literature derived epidemiological parameters. Our protocol is registered at osf.io/vp56f.

## Results

### Search results

**Fig. 1:**
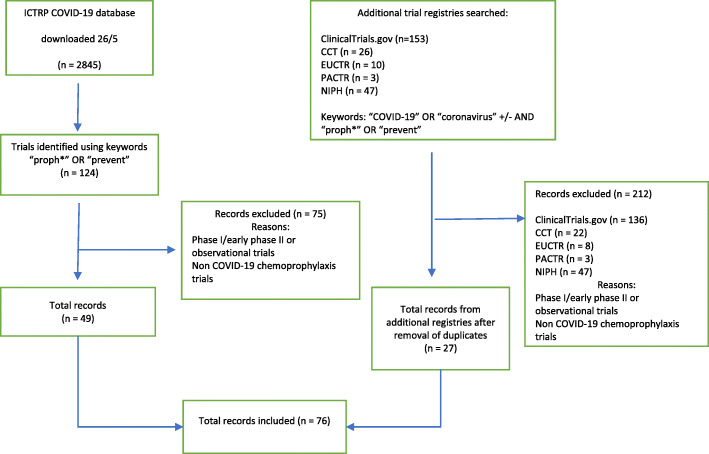
Flow diagram for inclusion and exclusion of chemoprophylactic trial protocols

The WHO ICTRP database search identified 124 trials of which 79 were excluded on title screening and review of trial components. After searching ClinicalTrials.gov, 17 additional studies were included. A search of the Chinese Clinical Trials Registry and EU Clinical Trials Register identified an additional four and two trials, respectively. No COVID-19 chemoprophylaxis studies were found on the NIPH or the PACTR. Six phase one and early phase two trials, and 3 observational trials were excluded. A total of 76 trials were deemed eligible for inclusion in this review (Fig. 1).

### Trial design

We found 76 interventional trial registrations, of which 63 (82.9%) were randomised and 13 (17.1%) non-randomised. Control groups assessing placebo or standard treatment were included in the design of 61/63 (96.8%) randomised trials and 9/13 (69.2%) non-randomised trials. Proposed enrolment for randomised trial registrations was 197,010 with median 600 (interquartile range (IQR), 236–1834). Non-randomised trial registrations proposed a total enrolment of 11,357 with median 400 (IQR, 300–1000). Cluster randomisation was specified as the randomisation method in 8 trial protocols, 7 of which were household (HH) PEP studies [12–18]. One long-term care facility (LTCF) trial proposed a cluster-randomised step wedge design, assessing HCQ in both elderly residents and HCWs [19].

PrEP was the approach in 55/76 (72.4%) studies with a total recruitment number of 180,848 (median, 440; IQR, 203 to 1500). PEP in household contacts comprised 16/76 (21.2%) studies with proposed recruitment of 20,951 participants (median, 1200; IQR, 360 to 2150). Four trials (5.3%) proposed PEP in non-HH contact studies (one LTCF, the others targeting HCW). We found only one trial registration, the CROWN CORONATION CQ trial, reporting an adaptive design which allowed for dose regimen arms to be added or removed [20]. No explicitly platform trials were identified.

The total estimated enrolment number across all trials was 208,367 people, ranging from 45 to 55,000 and with a median of 490 (IQR, 262 to 1710) and mean of 2742 (standard deviation, 8107). Five trials met our definition of a megatrial. The CROWN CORONATION trial aimed to randomise 55,000 people to one of four arms, assessing CQ in either a weekly, twice weekly, daily dose or placebo arm [20]. The COPCOV trial, aiming to enrol 40,000 HCW, is effectively two studies of 1:1 drug vs placebo, with CQ in Asia and HCQ in Europe [21]. The third largest trial assessed traditional Chinese medicine (TCM) against placebo and aimed to enrol 20,000 people from the general adult population in China [22]. Two other megatrials both assessed HCQ against either standard practice or placebo in HCW and aimed to enrol 15,000 and 10,990 people respectively [23, 24].

### Endpoints

Primary endpoints varied between trials, and protocols were not consistently clear as to whether the primary outcome required presence of COVID-19 symptoms as well as laboratory confirmation. PCR testing alone was specified as the diagnostic method in 35/76 (46.1%) studies, serological testing alone in 5 (6.6%) studies and 11 (14.5%) studies proposed both PCR and serological testing. Twenty two (28.9%) trial registrations did not specify or record the method of testing. Laboratory-confirmed SARS-CoV-2 infection was the primary outcome in 41/76 (53.9%) trials. Laboratory-confirmed infection including symptoms was the primary outcome in 32 (42.1%) trials. If trials did not address these endpoints as primary endpoints, they were often included as secondary endpoints. Only one trial included hospitalisation as a primary outcome. Other primary outcomes included presence of COVID-19 symptoms without laboratory confirmation, number of days off work due to illness and time until laboratory-confirmed infection. Mortality was a secondary outcome in 17/76 (22.4%) trials. PCR was used in 35/76 (46.1%) trials, serological testing in 5 (6.6%) trials and 11 (14.5%) trials used both testing methods. 22/76 (28.9%) trials registrations did not specify which test was used. Baseline serological testing was included in 20 of 54 (37%) HCW trials.

### Statistical power

Details of expected effect size were found in only four trial protocols. A trial comparing CQ and HCQ with placebo assumed a hazard ratio of 0.77 [21], while two HCQ trials assumed a hazard ratio of 0.7 [25] and 0.3 [26], respectively. A CQ trial assumed a hazard ratio of 0.8 [27].

**Fig. 2:**
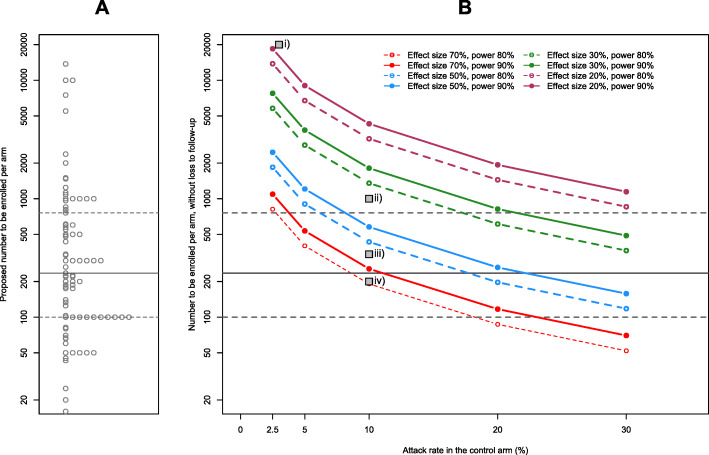
**A** Proposed trials by intervention arm size and **B** sample size required at a given power/effect size and estimated attack rate. Solid grey line indicates median arm size; dashed grey lines denote 25th and 75th percentiles

We compared the sizes of registered studies (per intervention arm) to estimates of the sample size required for a range of attack rates and effect sizes (with 1:1 randomisation; Fig. 2). As an example, for an attack rate of 10% [28] in the control arm, and a chemoprophylactic with relative risk reduction of 50%, a total sample size of approximately 870 people is required for 80% power. With these parameters, 50 trials (65.8%) could be underpowered at their current enrolment estimates. With a risk reduction of 30%, a total sample size of 2710 people is required at the same power level, meaning 74 trials (97.4%) could be underpowered. The four trials for which we found details on effect size sought are displayed in Fig. 2. Further details on outcomes and trial design can be found in Additional file 1.

### Populations

Of the 76 registered trials identified, 49 (64.5%) specifically included HCW in the trial target population. Fourteen of these HCW trials (18.4%) included LTCF workers in addition to hospital staff. Close contacts of COVID-19 cases were the target recruitment group in 11 (14.5%) trials. Five (6.6%) trials included both HCW and close contacts. Two studies, both evaluating traditional Chinese medicine (TCM), aimed to recruit from the general population [29, 30].

Three trials specifically targeted adults aged over 65 years (all in LTCF) [19, 31, 32]. The total recruitment number proposed for LTCF studies is 2736 with a median of 600 (IQR, 403 to 1265). We found a single paediatric trial, evaluating TCM [33]. Four (5.1%) trials evaluated chemoprophylaxis in patients with specific co-morbidities (Table 1). Pregnant women were specifically excluded in 56 (70.9%) trials.

**Table 1:** Summary of chemoprophylaxis study design features

Trial characteristics			Number of trials (%), n = 76	Number proposed to be enrolled (%); total, 208,367	Median (IQR)
**Design and intervention**	**Randomisation**				
	Randomised	Total	63(83)	197,010 (95)	600 (236–1834)
		Cluster randomised	8(11)	12,710 (6)	1575 (1050–2075)
	Non-randomised		13 (17)	11,357 (5)	400 (300–1000)
	**Approach**				
	PrEP		55(72)	180,848 (87)	440 (203–1500)
	PEP		21(28)	27,519 (13)	1000 (400–2000)
	**Blinding**				
	Single-blinded		10 (13)		
	Double-blinded^†^		36(47)		
	Open-label		28 (37)		
	Not stated		2 (3)		
	**Estimated sample size (N)**				
	≤ 299		18 (24)		
	300–499		20(26)		
	500–999		7 (9)		
	1000–4999		26 (34)		
	5000+		5 (7)		
	**Agent**				
	“Western” pharmaceutical agents		67 (88)		
	TCM/ayurvedic		9(12)		
**Population**	**HCW**		49 (64)	160,398 (77)	450 (350–1212)
	**Close contacts of COVID-19 patients**		11 (14)	11,326 (5)	600 (293–1410)
	**Both HCW and close contacts**		5 (7)	9625 (5)	2000 (1739–2486)
	**Older adults (65+ years)**		3 (4)	2736 (1)	600 (403–1265)
	**General population**		2 (3)	22,000 (11)	11,000 (6500–15,500)
	**Children (1–18 years)**		1 (1)	200 (<1)	N/A
	**Other/unspecified** (includes cancer, transplant and haemodialysis patients, cardiovascular/respiratory co-morbidities)		5 (7)	2132 (1)	200 (132–200)

^†^Also includes variants reporting higher levels of blinding

### Chemoprophylaxis candidates

“Western” pharmaceutical drugs were the proposed intervention in 67/76 (88.2%) registered trials. HCQ was assessed in 53/76 (70%) trials in at least one arm with regimens encompassing daily (200 to 600 mg) and less frequent dosing (200 mg weekly to 400 mg twice weekly). Eight HCQ trials conducted within-trial evaluation of different dosing regimens. Duration of PrEP treatment with HCQ ranged from 28 days to 6 months. HCW were the target group in 38/53 (71.7%) of HCQ trials. HCQ was assessed against both placebo/standard treatment and in combination and comparison to a number of agents. HCQ was used in combination with azithromycin, bromhexine, tenofovir/emtricitabine, vitamin D and zinc. Other trials compared HCQ to CQ, tenofovir/emtricitabine, lopinavir/ritonavir, umefenovir and azithromycin (Additional file 2). Of the 16 household PEP studies, 10 examined HCQ. Other PEP candidates were lopinavir/ritonavir, mefloquine, ivermectin, mycobacterium-w subcutaneous injection and TCM.

CQ was the proposed intervention in 4/76 (5.3%) trials, with two trials comparing CQ to placebo, one trial comparing CQ to HCQ and placebo, and one dose-ranging trial. CQ dosing regimens ranged from daily dosing of 150 to 250 mg or weekly doses of 300 to 500 mg, with treatment duration ranging from 10 to 12 weeks. Antiretroviral combination lopinavir/ritonavir was evaluated in 3/76 (3.9%) trials against HCQ or standard treatment. Other antiviral trials included interferons (3/76, 3.9%), isoprinosine (1/76, 1.3%), nitrozoxanide (1/76, 1.3%) and umefenovir (2/76, 2.6%, an antiviral licenced in China and Russia). TCMs were evaluated in 8 (10.5%) trials while 1 (1.3%) trial assessed ayurvedic agents. Further information on chemoprophylactic agents and recruitment numbers in individual trials are shown in Additional file 1.

## Discussion

This systematic review of COVID-19/SARS-CoV-2 chemoprophylaxis trial registrations during the first 21 weeks of the pandemic identified a number of challenges facing trialists in developing studies for emerging epidemic and pandemic diseases, as well as design aspects that support trial completion and clinical/public health relevance.

Adequate scepticism about expected attack rates and intervention effect size support powering studies sufficiently, so positive findings have a reasonable chance of being valid. Conversely, designing studies to convincingly rule out interventions is also important: insufficient study size, intervention timeliness relative to exposure or laboratory capacity for detection of specific endpoints can render negative findings inconclusive [34, 35].

We summarise some design considerations in Table 2.

**Table 2:** Considerations for design of chemoprophylaxis trials against emerging epidemic disease

Consideration	Comment
Study power	Trials should be powered based on adequate scepticism of expected effect size (recognising that modest effect size can have non-trivial utility in prophylaxis at scale against emerging infection) and attack rates (using early epidemic data and/or similar diseases/circumstances). For large studies premised on modest effect sizes, inclusion of interim analysis with suitable stopping rules can enable early termination if a prophylactic agent has a true high efficacy.
Population studied	Study population choices may be driven by the primary form of chemoprophylaxis efficacy under evaluation (see “Objective” below) and trial efficiency grounds. Demonstrating efficacy in a clinically relevant population should also drive study population e.g. selecting those at highest risk of severe outcomes if this is the primary study objective.
Objective: prevention of (symptomatic/asymptomatic) infection, symptomatic disease or severe disease and death	Infection is a prerequisite for disease and for onward transmission. If symptomatic disease is not substantially different from asymptomatic infection in health utility or transmission, then the higher frequency of any-infection over symptomatic infection may warrant consideration, including role in transmission interruption — a potential public health goal — and endpoint accrual rate. Conversely, severe outcomes such as hospitalisation, death and long-term sequelae of infection, while rare events represent clinically important outcomes. Covering different severities (infection, disease and severe disease between primary and secondary objectives of the study is likely to be necessary).
Endpoints: ever infected — serological markers of infection	Regular repeat or end of study serology may be an endpoint. Baseline serology may also be valuable in PEP and PREP studies: infection status at baseline may be used as e.g. a stratifying factor for pre-specified analysis. Serological sampling and frozen storage should be considered even if relevant serological assays are not available for a newly emerging disease epidemic at the start of the trial. Subsequent development or expansion of access could enable future analysis of stored samples.
Endpoints: current infection — samples for pathogen detection by (RT-)PCR or other methods	Testing for infection during a symptomatic episode is required if disease or severe disease is an endpoint. Frequent testing enables detection of asymptomatic or pre-symptomatic infection and conversion to disease. Sampling and frozen storage should be considered even if relevant pathogen detection assays are not available for a newly emerging disease epidemic at the start of the trial. Subsequent development or expansion of access could enable future analysis of stored samples [36].
Platform (adaptive) designs	Platform trial designs enable managed entry and exit of candidate chemoprophylactic agents as information accrues. Relatedly, randomisation need not be simple 1:1 against placebo but can be multi-arm and factorial.
Multi-centre/multi-site studies.	Geographical hotspots of transmission may move. Multi-centre/multi-site studies, including international collaborations, may facilitate endpoint accrual within a study. Alternatively, international collaborations may enable the use of equivalently designed chemoprophylaxis trials that support integrated analysis. For chemoprophylaxis trials, “multi-centre/multi-site” does not specifically refer to hospitals as the only setting for recruiting to studies.
Inter-epidemic research infrastructure preparedness: trials available early in epidemics	Research infrastructure for epidemic disease chemoprophylaxis enables rapid deployment when transmission is identified, supporting endpoint collation. For newly emergent diseases, catch-all registrations and approvals are unlikely to be feasible. Chemoprophylaxis research for outbreaks of known epidemic disease could be facilitated by specific prior trial registrations additional to the presence of general research infrastructure.
Pre- vs post-exposure chemoprophylaxis designs	Post-exposure trials would be expected to have higher attack rates in participants than pre-exposure trials, supporting trial efficiency, particularly in the absence of an uncontrolled generalised epidemic. An important trade-off in trial design is that time from disease (more specifically infectiousness) onset and diagnosis in the index case to initiation of PEP candidates in enrolled participants may enable infection to arise where the same agent may demonstrate efficacy if used pre-exposure. This biases towards the null. The same effect arises if index case and household contact have a common source of infection, or the “contact” is the index case infection source. PEP studies that have severe disease/death as an endpoint may also be testing early treatment effects of candidate drugs.
Therapeutics and anti-infective chemoprophylactics	Drugs for chemoprophylaxis do not need to be the same agents used therapeutically against severe endpoints in patients with the disease of interest; evidence from one scenario does not necessarily inform the other. For example, a drug with effects against viral replication and infection may have limited scope against inflammatory or autoimmune disorders in managing disease where an immunomodulatory drug may be most appropriate and vice-versa.

### Endpoints

Most COVID-19 chemoprophylaxis studies used endpoints of symptomatic disease; however, other endpoints warrant consideration in planning emerging infection chemoprophylaxis trials. Powering a study to look for infection, a more frequent endpoint, could support smaller, more efficient studies [37]. Infection endpoints may be ascertainable with serological monitoring bookending trial periods, alongside a regime of regular intra trial swabbing if there is appropriate laboratory capacity and the data collection burden on participants is minimal. If the ratio of true asymptomatic infections vs (mild/pre-)symptomatic cases is modest, the efficiency gain may not offset operational burden of repeat testing; however, the latter may be of value in an emerging infection. If an infection endpoint is used as the primary outcome, severe disease endpoints will be critical secondary endpoints to monitor. Powering a chemoprophylaxis trial for mortality may make chemoprophylaxis trials unfeasible. Including mortality as a secondary endpoint, which 21% of trials in this review do, supports monitoring cause-specific and all-cause mortality, including for safety. Similarly, powering a chemoprophylaxis trial for an endpoint of hospitalisation (as a marker for severe disease) will require a large sample size, and only one trial included this as a primary outcome.

Symptomatic disease without laboratory confirmation will reduce specificity of diagnosis. Laboratory-confirmed infection has value as an endpoint when testing is highly specific. Additionally, as a prerequisite to disease, laboratory-confirmed infection may contribute to the understanding of the use of chemoprophylaxis in preventing onward transmission. Serological endpoints with sufficient specificity can add value to trials by providing a cumulative incidence estimate, especially in situations where infection is subclinical, prompt diagnostic testing is unfeasible and/or sensitivity by virus/nucleic acid detection is imperfect — as may be the case for COVID-19 [38]. Endpoints which support trials reaching meaningful conclusions rapidly are of particular value during pandemics.

### Participants and pre- vs post-exposure

Most proposed studies target HCW and evaluate a PrEP approach. Methodologically, expected attack rates and propensity to actively engage in preventative epidemic research favour HCW involvement [39]. Variation in baseline immunity and attack rate in this highly exposed group should be taken into account when calculating sample size and analysing results. Studies show HCW seroconversion rates ranging from 1.6 [40] to 45% [41]; however, we found only 37% of HCW trials include baseline serological testing. Even if a study is established early in an epidemic, it does not preclude infection having already reached participants [42].

In contrast to HCW, there are relatively few chemoprophylaxis trials in population groups with a high-mortality risk — demonstrating chemoprophylactic efficacy in such groups will support external validity. Increasing age is the strongest risk factor for COVID-19 mortality [43]; however, only three trials specifically sought to recruit older adults, all residing in LTCF. Three trials target patients with chronic risk conditions (cardiac or respiratory disease, or requiring haemodialysis), while two target patients undergoing treatment for a cancer. We found that 70% of trial protocols specifically excluded pregnant women; however, protocols published online may not include the full exclusion criteria. This risk-benefit requires careful consideration with accumulating data from non-gravid populations, along with information on the potential for reproductive toxicity of the drug being studied [44].

The majority of PEP trials target household contacts. It is worth considering that at the point of enrolment in PEP trials, participants may have been infected by another source and/or infected days prior to exposure to the index case. Infection detection at the point of enrolment will be useful in this situation (even if randomisation balances distribution) and could be a component of endpoint definition/pre-specified analysis. Studies with disease (severity) endpoints may be measuring early treatment effects as well as infection-prevention effects and benefit from prospective design for this, including collaborative planning with therapeutics trialists.

### Size and power

We found many modestly sized studies registered. The US FDA considers that two small trials of *p* < 0.05 or one large trial with compelling evidence of efficacy would support licensure (in the absence of contradictory evidence) [45]. However, there are factors which make multiple small trials challenging. The effect size that can be anticipated in a trial with current prophylactic interventions is likely to be small (as proportional reduction in risk of being infected or diseased) — yet may have a large effect when deployed at scale, including the potential for indirect protection — so requiring a large sample size. Small low-powered trials which show a statistically significant result are less likely to reflect a true effect [46]. Trial heterogeneity may lead to inadequate strength of evidence — which might result from differences in study design and/or background risk of infection. Combining the results of multiple underpowered trials with participant level meta-analysis may be possible with full reporting of findings and data sharing. However, even if administratively feasible, it may not be reasonable if trials are too heterogeneous, particularly in intervention regimens, of which we found many variations.

In the small number of studies for which effect size assumptions were reported, these varied substantially. We conservatively estimated that more than 60% of trials risk being underpowered at an attack rate of 10%, even with substantial effect sizes, such as halving the infection or attack rate. In reality, these conditions may be difficult to achieve. A 10% attack rate means, for instance, that more than 3% of the participants will be infected each month if the study is conducted over 3 months. For lower monthly infection rates, longer study periods will be required, which might not be feasible. The large variation in trial size is reflective of the challenge of calculating appropriate sample sizes given the evolving epidemic dynamics. Lower event rates may be observed in older adults and other sheltering groups compared to those seen in HCW, resulting in an increase in sample size and time needed to enrol participants. Prevalence of circulating virus in different settings is also an important consideration. PrEP trials enrolling in regions where community transmission is suppressed means participants will have fewer episodes of exposure over the duration of a trial. Clinically meaningful chemoprophylactic effects against infectious diseases at the individual and population level and their impact on trial design warrant further exploration.

### Candidate agents and platform designs

HCQ was by far the most frequently registered chemoprophylactic agent, with 56 trials including it in at least one arm, at heterogeneous loading and steady-state doses. Data to date do not favour HCQ as a therapeutic [6, 47] and a PEP study showed no efficacy (though used a non-specific endpoint and allowed up to four days from exposure to drug administration) [34]. Additionally, unnecessary duplication of studies of the same agent increases waste. Emerging evidence regarding chemoprophylactic agent effectiveness in a rapidly evolving pandemic may require trials to adjust arms or terminate. While protocol amendments are readily feasible, adaptive and platform designs can account for this from conceptualisation and improve agility. We found evidence of adaptive design in a single registration (though cannot preclude that full protocols include this).

Our rapid systematic review of trial designs is limited to the information that is published on online protocols on selected international database sites, including those machine translated from Chinese to English. Available information is not standardised and some do not reveal important information regarding dosing regimen, effect size sought, blinding, allocation ratios and duration of treatment or follow-up. We did not attempt to scrutinise recruitment by participant ethnicity.

### Final considerations

We found that many registered COVID-19 chemoprophylaxis efficacy trials were underpowered to detect clinically meaningful protection at epidemiologically informed attack rates. Trials of chemoprophylactic agents are an important part of epidemic response if they can be started and completed during the early phases of epidemics, while vaccines are being developed. While vaccine development may take a long period of time and stretch over the duration of the actual outbreak, this has not been the case with COVID-19 vaccines. Within months, several vaccine candidates entered phase 2/3 clinical trials and enrolled tens of thousands of participants, and these vaccines are now being deployed at scale. The speed of vaccine development, compounded with the time taken to organise chemoprophylactic trials, has reduced interest in enrolling chemoprophylactic agents and marginalised these trials.

International coordination mechanisms and collaboration is required to support the design of feasible chemoprophylaxis trials, large enough to generate strong evidence, early on in an epidemic. Adaptive platform trial designs which allow structured entry and exit of candidate agents, as well as rapid stand-up of trial infrastructure in order to evaluate chemoprophylactic agents in an outbreak. Of pharmaceutical interventions in epidemics, chemoprophylaxis receives less attention than vaccines and therapeutics. The platforms and structures that have been developed in recent years for both vaccines and therapeutics do not yet exist to evaluate multiple chemoprophylactic agents in large scale, adaptive trials. These international coordination mechanisms should also be established for chemoprophylactic trials to ensure their rapid design and implementation in the early stages of an epidemic, enabling generation of high quality, widely applicable data.

## Supplementary Information

**Additional file 1.** Chemoprophylactic trial registration details. Description: Further details of each chemoprophylactic trial registration

**Additional file 2.** Chemoprophylactic trial agents and proposed recruitment. Description: Graph depicting individual trial agents and proposed enrolment numbers

## Data Availability

Data sharing is not applicable to this article as no primary datasets were generated or analysed during the current study.

## Abbreviations

SARS-CoV-2: Severe acute respiratory syndrome coronavirus 2
HCW: Healthcare workers
PrEP: Pre-exposure prophylaxis
PEP: Post-exposure prophylaxis
LTCF: Long-term care facilities
HCQ: Hydroxychloroquine
CQ: Chloroquine
WHO: World Health Organisation
ICTRP: International Clinical Trial Registry Protocol
NIPH: National Institute of Public Health
PACTR: Pan African Clinical Trial Registry
TCM: Traditional Chinese medicine
