# Supplementary material for: Chemoprophylaxis trial designs in epidemics: insights from a systematic review of COVID-19 study registrations
Source: Trials. 2021 May 29;22:370. doi: 10.1186/s13063-021-05323-4 (PMC8164073; doi:10.1186/s13063-021-05323-4)
Supplement: Supplementary file 2 — Additional file 2. Chemoprophylactic trial agents and proposed recruitment. Description: Graph depicting individual trial agents and proposed enrolment numbers [file 13063_2021_5323_MOESM2_ESM.docx]

***Figure 1:*** *Trials in HCW with planned enrolment less than 3000*

***Figure 2:*** *Trials in HCW with planned enrolment less than 3000*


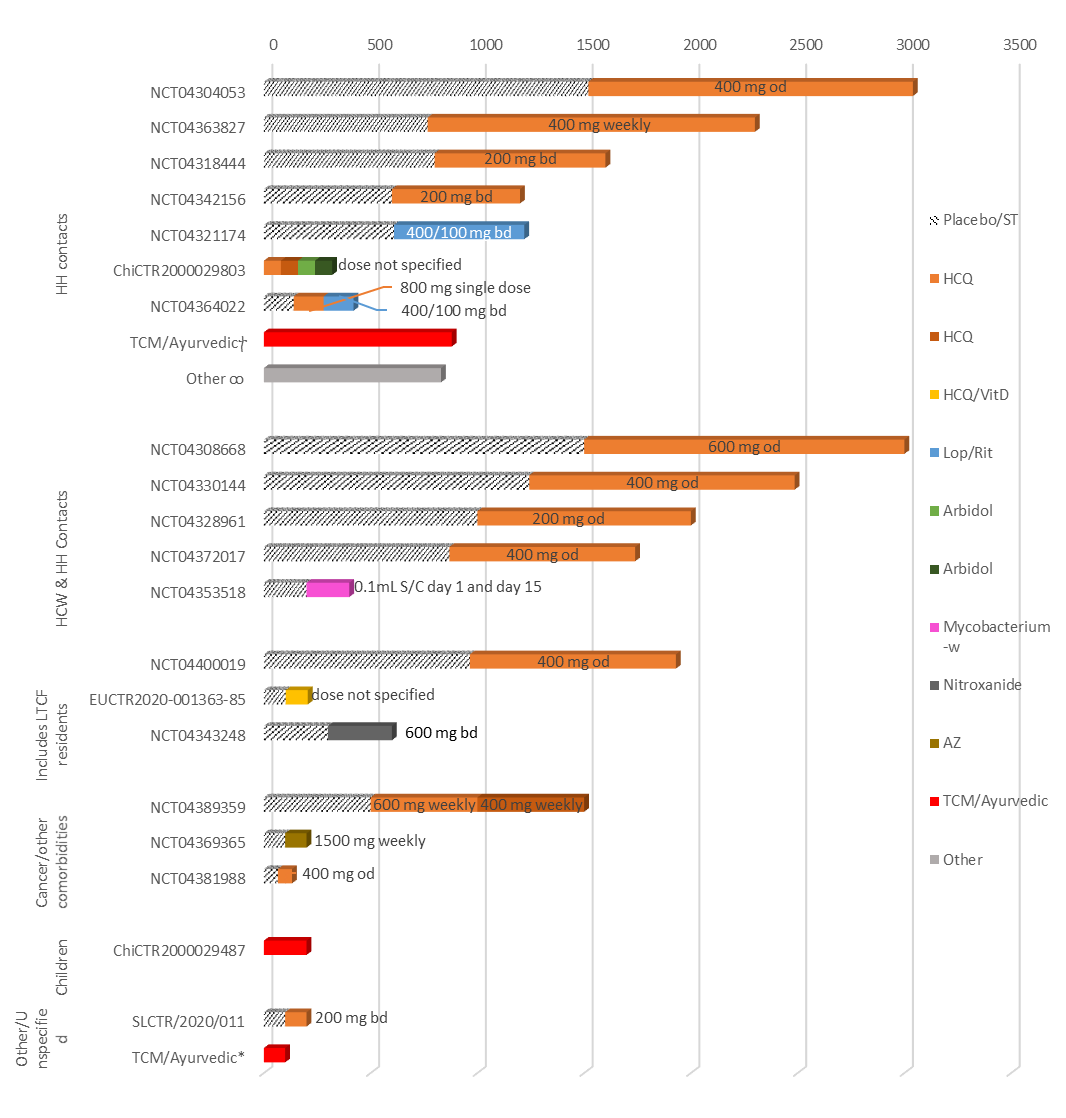

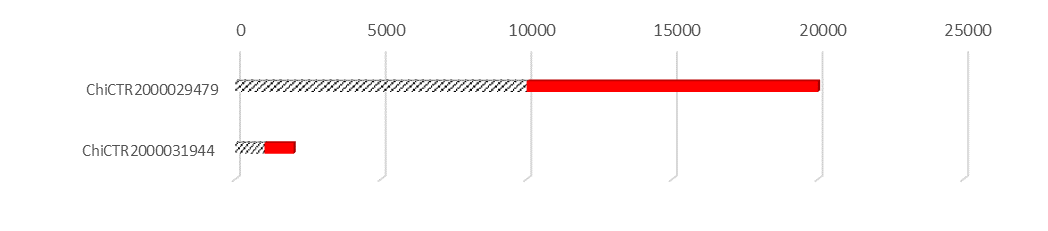


General adult population$\#$

Unspecified

**Figure 3:** Trials including non-HCW populations
